# Flares of Confusion: A Case Report of Uterine Leiomyoma and Angiomatosis Complexity on Imaging

**DOI:** 10.7759/cureus.50921

**Published:** 2023-12-21

**Authors:** Akram Al-Ibraheem, Marwah Abdulrahman, Ahmed Abdlkadir, Mohammad Abu Shattal, Maysa Al-Hussaini

**Affiliations:** 1 Nuclear Medicine and PET/CT, King Hussein Cancer Center (KHCC), Amman, JOR; 2 Diagnostic Radiology, King Hussein Cancer Center (KHCC), Amman, JOR; 3 Pathology and Laboratory Medicine, King Hussein Cancer Center (KHCC), Amman, JOR

**Keywords:** mri, uterine leiomyoma, uterine angiomatosis, synchronous tumors, pet imaging

## Abstract

Over the past decade, a series of rare and extraordinary uterine tumors have been discovered, with some featuring exceptionally uncommon tumor types. This highlights the growing recognition of these rare tumors due to evolutionary radiologic advancements. However, evaluating these patients requires adequate understanding to avoid misinterpretation and potential confusion with alternative differential diagnoses. This case is the first documented instance of two coexistent uterine benign tumors in a 45-year-old unmarried female patient. The patient's medical, gynecological, and surgical histories were unremarkable. Conventional abdominopelvic imaging via computed tomography (CT) and magnetic resonance imaging (MRI) revealed significant uterine expansion, indicating an atypical leiomyomatous or potentially leiomyosarcomatous mass. Subsequently, [^18^F]fluorodeoxyglucose ([^18^F]FDG) positron emission tomography (PET)/CT revealed hypermetabolic uterine enlargement, suggesting further evaluation. The patient underwent radical hysterectomy, and histopathological analysis revealed multiple uterine fibroids concurrently existing against the uterine angiomatosis. This case is the first documented instance of intricate pathological interplay within the uterus.

## Introduction

Uterine angiomatosis is an infrequent benign vascular tumor that was initially characterized through histopathological examination in 2017 [1]. Identification of this tumor can be difficult owing to its infrequent occurrence and absence of clearly defined morphological features. The presence of uterine fibroids alongside this tumor can introduce additional complexities to the diagnostic evaluation, thereby further complicating the assessment process. Uterine leiomyomas, a common condition in reproductive-aged women, may occasionally be present alongside other uterine tumors [2,3]. As a result, atypical radiological features can be observed, as observed in our specific case. Occasionally, some patients may encounter nonspecific abdominal pain, leading to delays in both radiological and gynecological evaluation [4]. It is important to acknowledge that the clinical and radiological characteristics of these tumors frequently exhibit similarities to those of other pathological conditions, resulting in diagnostic uncertainty [5].

In general, angiomatosis is not limited to a specific anatomical location but rather can present in various regions of the human body [6-9]. Within the female genital tract, this particular condition can manifest as discrete lesions, occur alongside other hemangiomas affecting the skin or other organs, or be a constituent of intricate syndromes such as the Klippel-Trénaunay-Weber syndrome [10].

The primary objective of our case report is to present an extraordinary and illustrative case, with the intention of enhancing awareness regarding the unique radiological characteristics of this rare pathological interaction. Therefore, abdominal radiologists are expected to consider this condition as a possible diagnosis when they encounter similar radiological presentations.

## Case presentation

A 45-year-old unmarried woman presented with a three-month history of abdominal distention and periumbilical pain. Her abdominal pain gradually increased and became more generalized through the last month of her presentation. Upon presentation to our cancer center, she reported an unremarkable past medical, surgical, and gynecological history. She also reported regular menstrual cycles. When asked about chronic medication use, she denied chronic medication use and had never taken any form of oral contraceptive pills. On physical examination, the patient was found to have significant abdominal distension, abdominal resonance, and periumbilical tenderness.

**Figure 1:**
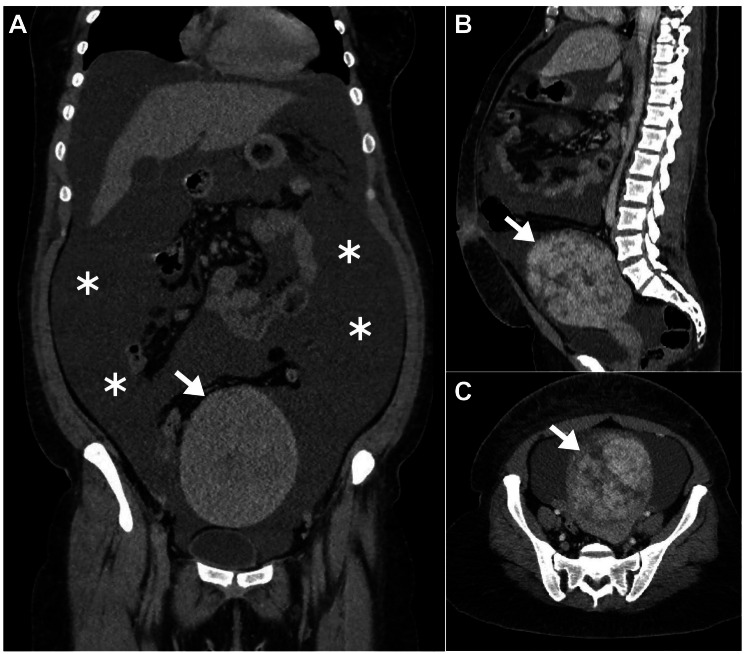
Abdominopelvic CT. (A) Coronal abdominal CT revealed massive ascites (asterisks) with evidence of a large uterine mass budding toward the abdominal cavity (arrow). (B) Sagittal CT and (C) axial CT revealed evidence of massive uterine enlargement with heterogeneous density (arrows). CT: computed tomography

Initial diagnostic imaging included abdominopelvic computed tomography (CT), which revealed well-defined massive ascites in concordance with a massive heterogeneous density of the uterine mass reaching the level of the umbilicus (Figure 1).

**Figure 2:**
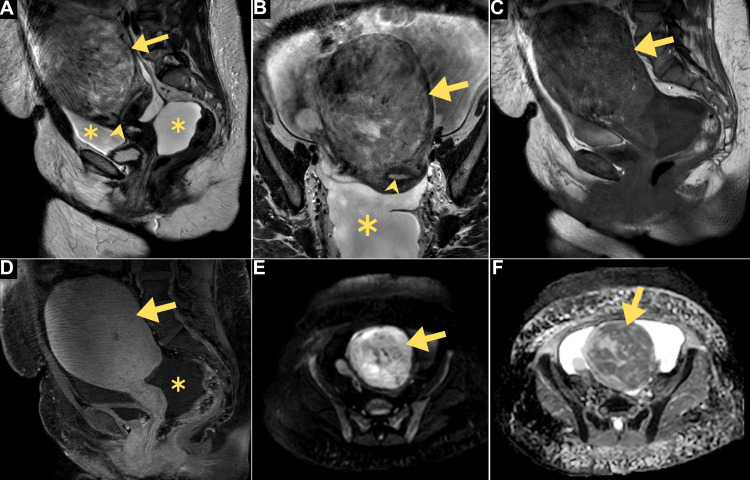
Multiplanar pelvic MR images. (A) Sagittal and (B) coronal T2-weighted images depicting a ventral uterine mass (arrows) expanding the anteverted uterus, compressing the endometrium (arrowhead), and demonstrating predominant tumoral hyperintensity. (C) Sagittal T1-weighted images precontrasted and (D) postcontrasted reveal hypointense signals and homogeneous hyperenhancement. (E) Axial diffusion-weighted imaging showing a hyperintense mass that appeared hypointense on the apparent diffusion coefficient map (F), indicating restricted diffusion. Notably, there is a conspicuous presence of surrounding ascites (denoted by asterisks). MR: magnetic resonance

Concurrently, pelvic magnetic resonance imaging (MRI) was performed and revealed a 13.5 cm ventral intramural uterine mass demonstrating heterogeneous intensity with features suggestive of atypical leiomyomatous or leiomyosarcomatous pathologies (Figure 2).

**Figure 3:**
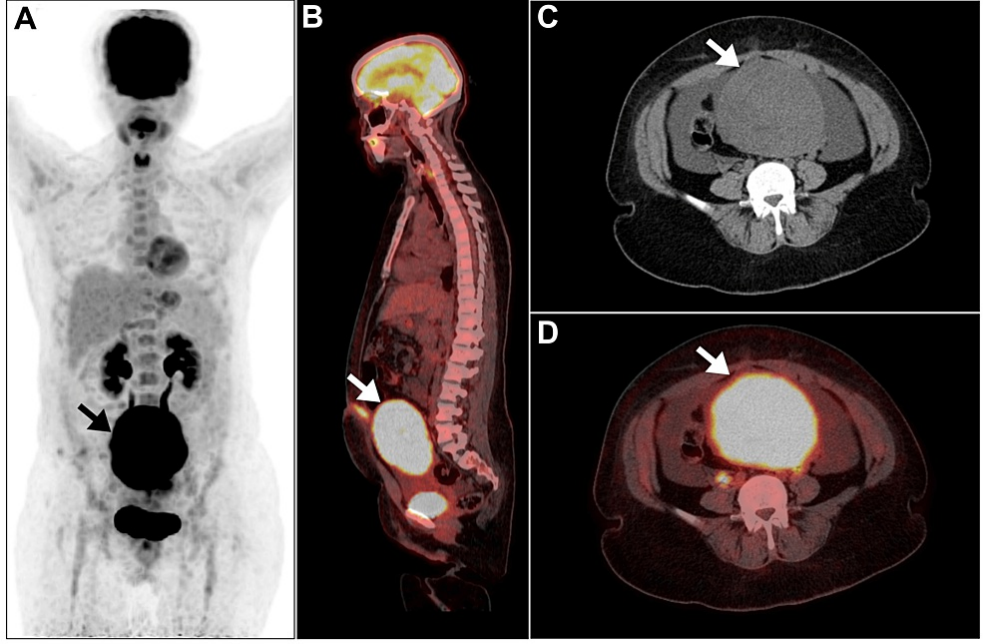
[18F]FDG PET/CT image. (A) MIP revealing evidence of a large hypermetabolic uterine mass (arrow). (B) Sagittal fused [^18^F]FDG PET/CT image demonstrating high hypermetabolic activity involving the enlarged uterus and extending to the umbilicus, with a maximum SUVmax of 26.3 (arrow). (C) Axial CT image and (D) axial PET/CT image demonstrating massive uterine enlargement with intense [^18^F]FDG activity (arrow). [^18^F]FDG: [^18^F]fluorodeoxyglucose; PET: positron emission tomography; CT: computed tomography; MIP: maximum intensity projection; SUVmax: maximum standardized uptake value

At that time, laboratory analysis was carried out and included a complete blood count, bleeding profile, liver function test, thyroid function test, pregnancy test, renal function test, liver virology profile, fasting glucose level, and electrolytes. In addition, carcinoembryonic antigen and carbohydrate antigen (CA), specifically CA 125, CA 153, and CA 199, were also detected. All these tests were unremarkably normal, except for the mild elevation of CA 153, which was 36.8 U/ml (exceeding the upper normal range of 34.5 U/ml), and the notable increase in CA 125, which was 159 U/ml (exceeding the normal reference of 35 U/ml). Subsequently, [^18^F]fluorodeoxyglucose ([^18^F]FDG) positron emission tomography (PET)/CT was performed and revealed well-defined intense hypermetabolic activity involving the enlarged uterus and extending to the umbilicus (Figure 3).

**Figure 4:**
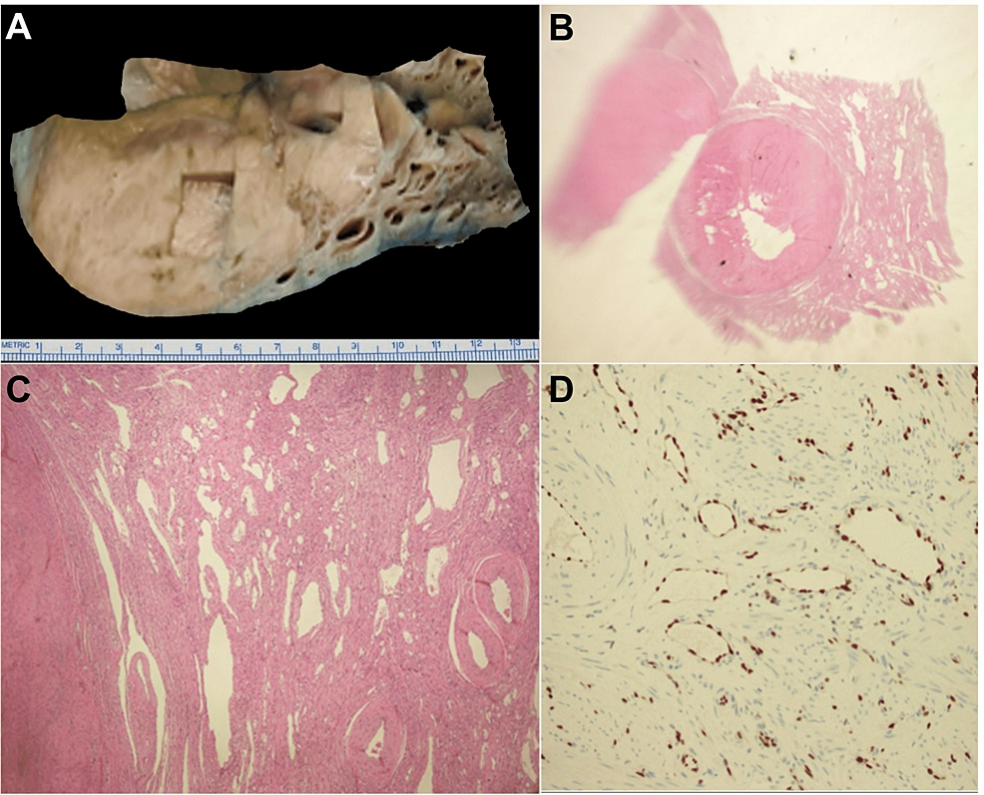
Histopathologic examination of the resected uterus. (A) Gross section of the resected uterus demonstrating a sponge-like appearance in the outer half of the myometrium. (B) A scanning power magnification of the uterine leiomyoma and (C) high-power magnification of the uterine leiomyoma denoting evidence of ectatic vessels in the surrounding myometrium. (D) Immunohistochemical staining for the ERG revealed positive expression at the site of the endothelium of the ectatic vessels, consistent with the diagnosis of uterine angiomatosis. ERG: erythroblast transformation-specific-related gene

The patient's clinical condition was discussed in a multidisciplinary clinic, and it was decided that surgical intervention should be pursued with radical hysterectomy. During surgery, 9 liters of ascites were removed. Histopathologic examination of the resected uterus revealed evidence of several leiomyomatous buddings against a background of predominant angiomatosis (Figure 4).

These predominant ectatic vessels were also found within both the adnexa and the cervix, reflecting angiomatosis. To date, the patient has been monitored and followed up regularly and has remained asymptomatic and disease-free for the past six months, up until the conclusion of her follow-up.

## Discussion

Angiomatosis is a diffuse vascular lesion that involves a large segment of the body in a contiguous fashion and involves multiple tissues (e.g., the uterus, adnexa, cervix, vagina, etc.) in different planes [1]. Once predominantly enlarged, angiomatosis can cause notable swelling and distension and may exert pressure on adjacent organs [6]. It can also present with nonspecific gynecologic symptoms, such as uterine bleeding [1]. The main pitfall when evaluating angiomatosis is the fact that it can present as diffuse infiltration, providing a pseudomedical picture [6]. Histopathological diagnosis of angiomatosis is important due to its high recurrence rate [6]. To date, there are no reported instances where angiomatosis can transform into malignant neoplasms [11]. Nonetheless, regular and routine follow-up is needed given the rarity of this tumor type and its recurrence potential.

Retrospective examination of the whole set of biochemical and radiological results can help us reach diagnostic insights. Typically, uterine leiomyomas present as well-defined masses demonstrating homogeneous density and intensity on CT and MRI, respectively [12]. On the other hand, uterine leiomyosarcomas tend to have ill-defined borders and a heterogeneous pattern [13]. Our case was visualized with well-defined margins but a heterogeneous pattern and was therefore regarded as atypical and confusing between the aforementioned entities. However, both leiomyomas and leiomyosarcomas rarely manifest as massive abdominal ascites [14,15]. This is usually correlated with massive uterine enlargement exceeding 20 cm in the maximum dimension [14,15].

Our radiologic findings, when paired with biochemical analyses performed shortly after surgery, can provide insight into the uterine complexity encountered in our patient. The elevated CA 125 and CA 153 levels lie far beyond the typical level of uterine leiomyoma for a given uterine size and are still lower than the typical expression levels used to indicate malignancy [16]. Notably, the patient's uterine mass was visualized using [^18^F]FDG PET/CT, which revealed intense [^18^F]FDG uptake with a maximum standardized uptake value (SUVmax) of 26.3. There was no hypermetabolic deposition within the massive ascites of the peritoneum or lymph nodes. Therefore, the [^18^F]FDG PET/CT report was consistent with atypical leiomyoma rather than leiomyosarcoma. Typically, leiomyomas attain moderate levels of [^18^F]FDG uptake [17]. Angiomatosis, on the other hand, has been studied at other body locations and has been shown to demonstrate moderate [^18^F]FDG expression [6-9]. The causal factor for such intense [^18^F]FDG activity seems to be the metabolic potentiation effect of leiomyoma on the basis of the background of angiomatosis, giving rise to high [^18^F]FDG uptake derived from the combination of both benign tumors potentiating [^18^F]FDG expression. Therefore, this case report aims to showcase the significance of collaborative and interdisciplinary team analysis in the evaluation of uterine complexity. In our perspective, this case will open a new landscape for researchers to examine patterns of uptake for hybrid and/or complex tumors.

## Conclusions

Physicians need to be aware of such uterine complexities. Unraveling uterine complexity proves to be a formidable undertaking when solely relying on clinical, radiological, or biochemical indicators. Consequently, the manifestation of atypical features should always prompt interdisciplinary consultation, intermodality correlation, and biochemical evaluation.
